# Inhibitory Effect of Periodontitis through C/EBP and 11β-Hydroxysteroid Dehydrogenase Type 1 Regulation of Betulin Isolated from the Bark of *Betula platyphylla*

**DOI:** 10.3390/pharmaceutics14091868

**Published:** 2022-09-05

**Authors:** Eun-Nam Kim, Gil-Saeng Jeong

**Affiliations:** College of Pharmacy, Chungnam National University, Daejeon 34134, Korea

**Keywords:** periodontitis, betulin, human periodontal ligament cells, 11β-hydroxysteroid dehydrogenase type 1, inflammation, osteogenic induction

## Abstract

Periodontitis is an infectious inflammatory disease of the tissues around the tooth that destroys connective tissue and is characterized by loss of periodontal ligaments and alveolar bone. Currently, surgical methods for the treatment of periodontitis have limitations and new treatment strategies are needed. Therefore, this study evaluated the efficacy of the compound betulin isolated from bark of *Betula platyphylla* on the inhibition of periodontitis in vitro and in vivo periodontitis induction models. In the study, betulin inhibited pro-inflammatory mediators, such as tumor necrosis factor, interleukin-6, inducible nitric oxide synthase, and cyclooxygenase-2, in human periodontal ligament cells stimulated with *Porphyromonas gingivalis* lipopolysaccharide (PG-LPS). In addition, it showed an anti-inflammatory effect by down-regulating 11β-hydroxysteroid dehydrogenase type 1 and transcription factor C/EBP β produced by PG-LPS. Moreover, PG-LPS inhibited the osteogenic induction of human periodontal ligament cells. The protein and mRNA levels of osteogenic markers, such as inhibited osteopontin (OPN) and runt-related transcription factor 2 (RUNX2), were regulated by betulin. In addition, the efficacy of betulin was demonstrated in a typical in vivo model of periodontitis induced by PG-LPS, and the results showed through hematoxylin & eosin staining and micro-computed tomography that the administration of betulin alleviated alveolar bone loss and periodontal inflammation caused by PG-LPS. Therefore, this study proved the efficacy of the compound betulin isolated from *B. platyphylla* in the inhibition of periodontitis and alveolar bone loss, two important strategies for the treatment of periodontitis, suggesting the potential as a new treatment for periodontitis.

## 1. Introduction

Periodontitis is a chronic disease that leads to the destruction of the periodontal ligaments and loss of the alveolar bone at the same time as inflammation of the tooth supporting structures. Therefore, it is an important treatment strategy to suppress the inflammation of periodontal tissue and the loss of periodontal tissue and alveolar bone due to inflammation [1]. In the early stage of periodontal disease, many bacteria exist, which converts the lesion to inflammation along with proliferation, and the microbial flora community in the periodontal pocket formed by periodontal tissue damage progresses further, causing destruction of periodontal tissue [2].

Among the many enzymes involved in the biosynthesis and catabolism of glucocorticoids, 11β-hydroxysteroid dehydrogenase type 1 (11β-HSD1) converts cortisone to cortisol to regulate glucocorticoid levels, and excessive glucocorticoids cause several metabolic disorders such as diabetes, obesity, and periodontitis [3,4,5]. It has been suggested that 11β-HSD1 plays a pivotal role in the intracellular regulation of glucocorticoids, and the regulation of 11β-HSD1 is known as an important therapeutic strategy in metabolic disorders [6,7]. According to previous research reports, 11β-HSD1 deficient mice exhibited obesity, stress, and resistance to insulin due to a weakened glucocorticoid-induced response, and 11β-HSD1 inhibitors are currently being developed as therapeutic agents [8,9]. However, the role of the regulation of 11β-HSD1 in periodontitis is still being studied.

The expression of 11β-HSD1 in several inflammatory diseases, including rheumatoid arthritis, has been demonstrated in previous studies, suggesting that increased 11β-HSD1 plays a role in chronic inflammation [10,11]. Therefore, to demonstrate the relevance of 11β-HSD1 in chronic periodontitis, human periodontal ligament cells stimulated with *Porphyromonas gingivalis*-LPS and an in vivo in vivo model of periodontitis induction were used in the study. A recent study reported that the gingival tissue of chronic periodontitis patients expressed higher 11β-HSD than normal, suggesting that increased 11β-HSD1 plays a role in chronic gingivitis [12]. In addition, it has been reported that the synthesis of glucocorticoid by 11β-HSD1 overexpressed in osteoblasts improves bone resorption, leading to osteopenia and ultimately osteoporosis [13]. This suggests the possibility of recovery of lost alveolar bone through the recovery of periodontal ligament cells, which is a treatment strategy for periodontitis, and inhibition of 11β-HSD1 in the process of differentiation into osteoblasts.

*Betula platyphylla* is distributed in Korea, Japan, China and Siberia, and the bark of *B. platyphylla* has been used as a folk medicine in Asia for arthritis and skin diseases [14,15]. As the composition of birch, diarylheptanoids and arylbutanoids have been analyzed and identified as major constituents in the bark, and phenolic compounds, such as diarylheptanoids and arylbutanoids, have been reported in the outer and endothelium, along with terpenoids containing botulin [16,17,18,19]. The main component of *B. platyphylla* Bark, botulin (lup-20(29)-ene-3β, 28-diol), has a triterpene structure, it is known that the secondary hydroxyl group of C-3, the primary hydroxyl group of C-28, and the double CeC bond of C-20 exhibit structural activity, Also, it can be converted to betulinic acid either synthetically or by biotransformation [20]. According to previous studies, betulin can reduce LPS-induced inflammation and stress, and has been reported to have a wide range of biological and pharmacological effects on various cell lines such as hepatocyte protective effect and anti-HIV effect [21,22,23,24]. However, despite the broad pharmacological activity of betulin, the pharmacological efficacy of betulin for periodontitis has not yet been reported. Currently, the most effective treatment method for periodontitis is to combine drug treatment with surgical operation. However, these treatment methods have a poor treatment prognosis and may cause pain and side effects in patients. Therefore, in this study, the pharmacological effect of betulin isolated from bark of *B. platyphylla* in human periodontal ligament (HPDL) cells stimulated with PG-LPS and in an in vivo periodontitis-induced animal model was investigated.

## 2. Materials and Methods

### 2.1. Chemicals and Reagents

The minimum essential medium alpha (α-MEM) and fetal bovine serum (FBS) were purchased from Welgene Bioscience (Daegu, Korea) and used for cell culture. Moreover, trypsin-ethylene diamine tetra acetic acid (EDTA) and penicillin used for cell subculture were purchased from Gibco (Grand Island, NY, USA). 3-(4,5-Dimethylthiazol-2-yl)-2,5 diphenyltetrazoliumbromide (MTT) for measuring cell viability and Alizarin Red S, Dimethyl Sulfoxide Dimethyl Sulfoxide (DMSO) for measuring osteoblastic differentiation capacity were purchased from Sigma Aldrich (Saint Louis, MO, USA). Lipopolysaccharide isolated from *P. gingivalis* (PG-LPS) used for periodontitis induction was purchased from Invivo Gen (San Diego, CA, USA). The primary antibody of 11β-HSD1, OCN and RUNX2 used in Western blot assay was purchased from Invitrogen (Carlsbad, CA, USA), and antibodies of p-C/EBP β, C/EBP β, iNOS, COX-2, and β-actin were purchased from Santa Cruz Biotechnology Inc. (Dallas, TX, USA). TRIzol reagent was purchased from Thermo Fisher Scientific, Inc. (Waltham, MA, USA). In addition, rabbit and mouse secondary monoclonal antibodies were purchased and used from Santa Cruz Biotechnology Inc. and Cell Signaling Technology (Danvers, MA, USA). HPLC grade water, acetonitrile, and methanol used for HPLC-DAD analysis were purchased from Thermo Fisher Scientific, Inc. *n*-Hexane, ethyl acetate, *n*-butanol, and chloroform were obtained from Daejung Chemicals and Metals Co., Ltd. (Siheung, Korea).

### 2.2. Plants Materials

The bark of *B. platyphylla* was obtained in Gangwon Province Inje (Korea), and it was identified by Professor Gil-Saeng Jeong of the College of Pharmacy at Chungnam National University. A voucher specimen (CNU-211105) has been deposited in the natural products laboratory, College of Pharmacy, Chungnam National University, (Daejeon, Korea).

### 2.3. Extraction and Isolation

Dried bark of *B. platyphylla* (685.4 g) was immersed in 70% EtOH for 24 h, heated to extract under reflux for 2 h, and then evaporated with a rotary vacuum concentrator to obtain an EtOH extract (140.2 g). The extraction yield was confirmed to be about 7.8%. Then, after suspended the EtOH extract with distilled water followed by liquid-liquid separation with CHCl_3_ (38.7 g), *n*-butanol (70.5 g), and water (34.4 g). Among them, the CHCl_3_ (38.7 g) fraction was subjected to silica gel column chromatography with CH_2_Cl_2_–MeOH–H_2_O (9:3:0.1) to obtain 11 fractions (Fr.1~11). After that, Fr. 4 (554 mg) was again subjected to column chromatography with CH_2_Cl_2_-MeOH (15:1, 3:1, 1:1) to obtain the lower 8 fractions (Fr.4-1~4.8). After that, fraction 4-4 (218.4 mg) was purified with a gradient solvent system of MeOH-H_2_O (90% MeOH–80% MeOH for 40 min) using semi-preparative RP HPLC to obtain 82.4 mg of recrystallization. Then, as a result of comparing the recrystallized structure with the previously reported literature by nuclear magnetic resonance (NMR, JNM-ECZR 500 MHz, Tokyo, Japan) [25], it was identified as betulin, and its NMR spectrum is as follows.

Betulin (C_30_H_50_O_20_): white needles, ^1^H NMR (500 MHz, CDCl_3_) δ: 0.75, 0.81, 0.96, 0.97, 1.04 (3H each, s, 5 × CH_3_), 1.66 (3H, s, H–30), 3.16 (1H, dd, *J* = 10.5, 5.0 Hz, H–3), 3.33 (1H, d, *J* = 11.0 Hz, H–28), 3.80 (1H, d, *J* = 11.0 Hz, H–28), 4.57 (1H, br s, H–29), 4.70 (1H, br s, H–29). ^13^C NMR (500 MHz, CDCl_3_) δ:14.8 (C–27), 15.5 (C–24), 16.0 (C–25), 16.2 (C–26), 18.4 (C–6), 19.3 (C–30), 21.0 (C–11), 25.3 (C–12), 27.1 (C–15), 27.5 (C–2), 28.1 (C–23), 29.3 (C–16), 29.8 (C–21), 34.1 (C–22), 34.3 (C–7), 37.2 (C–13), 37.4 (C–10), 38.8 (C–4), 39.0 (C–1), 41.0 (C–8), 42.8 (C–14), 47.9 (C–18), 48.0 (C–17), 48.8 (C–19), 50.5 (C–9), 55.4 (C–5), 60.7 (C–28), 79.1 (C–3), 109.8 (C–29), 150.5 (C–20).

### 2.4. Protein Analysis Using Western Blot Analysis

For protein analysis, the harvested HPDL cells were lysed with radioimmunoprecipitation assay (RIPA) buffer at refrigerated temperature for 30 min and then centrifuged at 12,000 rpm for 20 min. After quantification of the centrifuged supernatant by Bradford assay, 20 μg of each was loaded onto SDS (8–12%) polyacrylamide gel and electrophoresed. After transferring the protein electrophoresed on the gel to a PVDF (polyvinylidene difluoride) membrane, the membrane was blocked with 5% (*w*/*v*) non-fat dried milk at room temperature for 2 h. After washing each membrane with in Tris buffer 10 mM (pH 8.0, 150 mM NaCl), Incubate with the primary antibody monoclonal antibody overnight at 4 °C for 24 h at room temperature, followed by washing with Tris buffer. Then, sequentially, anti-rabbit or anti-mouse (Santa Cruz Biotechnology Inc., Dallas, TX, USA) was incubated with the secondary antibody for 2 h and washed with Tris buffer. After the antibody reaction was completed, the membrane was reacted with an enhanced chemiluminescence (ECL) solution before blots were taken using Healthcare Life Science ECL-plus (Tokyo, Japan) and normalized to the intensity level of β-actin.

### 2.5. Analysis of Gene Level Using RT-qPCR

To evaluate the mRNA level of each gene, Cells were lysed in TRIZOL reagent (Bioneer, Daejeon, Korea). Then, RNA was reverse transcribed using the RT PreMix kit (Enzynomics, Daejeon, Korea) and PCR amplified in the DNA Engine Opticon 1 continuous fluorescence detection system (MJ Re-search, Waltham, MA, USA) using SYBR Premix Ex Taq. Each PCR reaction was performed using the fol-lowing conditions: 95 °C 30 s, 60 °C 30 s, 72 °C 30 s, and plate read (detection of fluores-cent product) for 40 cycles followed by 7 min of extension at 72 °C. Melting curve analysis was performed to characterize the dsDNA product by slowly raising the temper-ature (0.1 °C/s) from 60 °C to 95 °C with fluorescence data collected at 0.2 °C intervals. mRNA levels of genes were normalized to Gapdh. The gene expression was calculated us-ing the following equation: Gene expression = 2^−ΔΔCT^, where ΔΔCT = (CT Target − CT gapdh). Primers for each gene used in this study were provided by Biomedic (Bucheon, Korea), and the sequences of the primers are presented in Table 1.

### 2.6. Mineralization Assay

The HPDL cells were seeded in 6-well plates at a density of 1 × 10^4^ cells/well. After 3 days, cells were cultured for osteogenic induction in osteo-induction medium (OIM) containing 50 μg/mL ascorbic acid and 10 mM β-glycerophosphate culture for 14 days. Mineralization assay was analyzed after 14 days. The HPDL cells were rinsed with PBS, fixed with 4% paraformaldehyde for 10 min and added with 1% alizarin red for another 30 min. Nonspecific staining was removed by several wash with PBS. The staining was extracted from the cell matrix by incubation with 10% cetylpyridinium chloride in 10 mM sodium phosphate (pH 7.0) for 10 min. The alizarin red concentration absorbance was measured at 560 nm using a multifunctional microplate reader (M1000 Pro, TECAN, Männedorf, Switzerland).

### 2.7. Periodontitis Induction Model Using PG-LPS

To evaluate the periodontitis inhibitory effect of betulin, the experiment was conducted by dividing the groups as follows. Sprague-Dawley rats (8 weeks old) were acclimatized for 1 week and then divided into 5 groups as follows: (1) Control (PBS orally administrateion), (2) PG-LPS (6 day induced, needle injection), (3) PG-LPS (6 day induced) + betulin 12.5 (12.5 mg/kg for 8 days, orally administrateion), (4) PG-LPS (6 day induced) + betulin 25 (25 mg/kg for 8 days, orally administrateion); and (5) PG-LPS (6 day induced) + betulin 50 (50 mg/kg for 8 days, orally administrateion), For anesthesia of experimental animals, isoflurane (Hana Pharm. Co., Ltd., Gyeonggido, Korea) was purchased and used. For PG-LPS, periodontitis was induced by injecting PG-LPS at a concentration of 10 mg/mL daily between the maxillary first and second molars. The entire experimental process was carried out with the approval of the Institutional Animal Care and Use Committee (IACUC) of Keimyung University Laboratory Animal Research Center (Permission No. KM2020-014) and the Animal Experiment Ethics Committee of Chungnam National University (Permission No. 2022203C-CNU-001).

### 2.8. Analysis of Periodontal Tissue Using Micro-CT Images

From the extracted periodontal tissue, two rectangular volumes (400 µm width × 500 µm thickness × 1400 µm height) of alveolar bone next to the mesial and distal root were analyzed for bone density analysis (Pixil size 10 μM). To measure bone mineral density (BMD) from the extracted periodontal tissue, the direction of the periodontal tissue was changed to a coronal section, and raw data obtained from Micro-CT was loaded into CTAn. After that, the region of interest (ROI) was analyzed by interpolation of the alveolar bone excluding the root of the cement-enamel joint, and the image was transmitted using VGStudio MAX 1.2.1 software (Volume Graphics, GmbH, Heidelberg, Germany). Data were analyzed as mean ± SD and one-way ANOVA in SPSS Statistics (Armonk, NY, USA).

### 2.9. Histological Staining

Another series of experiments was conducted for histological examination. After euthanasia, the extracted rat maxillary was perfused with a 10% formaldehyde neutral buffer (Sigma-Aldrich, St. Louis, MO, USA). Then the maxillary bone was excised and fixed in 10% formaldehyde neutral buffer for 3 days at 4 °C. The bones were decalcified for 24 h at 4 °C in K-CX (Falma, Tokyo, Japan), a rapid demineralization solution, followed by conventional dehydration and paraffin embedding. After cutting into 5 μm-thick sections, the specimen was deparaffinized and stained with hematoxylin-eosin (H&E). Thereafter, the tissue infiltrated area in dark purple in a certain area of the specimen was observed using a fluorescence Olympus IX microscope 71-F3 2PH (Tokyo, Japan). The observed infiltration sites were measured in areas of standardized rectangular slides (800 µm width × 800 µm length) in terms of tension and compression using ImageJ (National Institute of Health, Bethesda, MD, USA), and after drawing, using the sum of the areas, each of the total infiltration areas of the sample was calculated.

### 2.10. Goldner’s Masson Trichrome Staining

Goldner’s Masson Trichrome staining was performed to evaluate the effect of betulin on periodontal fibrous tissue formation. For histological examination, the extracted rat maxilla after the end of the experiment was fixed in 10% neutral formalin solution for 2 days. After that, the samples of each group were decalcified by the formic acid sodium citrate method, embedded in paraffin, and tissue sections were prepared with a thickness of 4 μm, and the area of a rectangular slide (500 μm width × 400 μm length) was measured. Subsequently, Goldner’s Masson Trichrome staining was performed, and the formation of periodontal fibrous tissue lost by PG-LPS was evaluated through an optical microscope Olympus IX microscope 71-F3 2PH (Tokyo, Japan).

### 2.11. ELISA Assays

After the end of the experiment, rat blood samples were collected from the jugular vein and centrifuged at 3000 rpm per minute for 10 min at 4 °C. Serum was extracted, stored in plastic tubes, and frozen at −20 °C to assess the levels of IL-1β, IL-6, and TNF-α. After that, the levels of IL-1β, IL-6, and TNF-α from the isolated serum were analyzed by enzyme-linked immunosorbent assay technology using a commercial kit (R&D Systems, Minneapolis, MN, USA) according to the manufacturer’s recommendations. Cytokines in each serum were measured using a microplate spectrophotometer at 450 nm.

### 2.12. Statistical Analysis

To confirm the reproducibility of the experiment Each and every experiment was performed in triplicate and analyzed as mean ± standard deviation (S.D.). Statistical analysis was performed from the analyzed values using SPSS Statistics 19.0 software (Armonk, NY, USA). Differences between groups were analyzed by one-way analysis of variance (ANOVA) followed by Tukey’s test or Student’s *t*-test. Statistical significance was judged as *p* value < 0.05.

## 3. Results

### 3.1. Quantitative Analysis of Betulin in Betula platyphylla Bark

**Figure 1:**
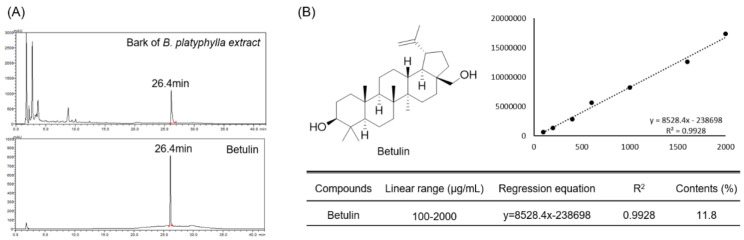
Quantitative analysis of Betulin in bark of *B. platyphylla* extract. And analysis of bark of *B. platyphylla* extract and betulin using HPLC DAD (**A**). Diode array detector wavelength (203 nm). Confirmation of linearity of isolated betulin and analysis of betulin content in bark of *B. platyphylla* extract (**B**).

The content of betulin contained in *B. platyphylla* bark was quantitatively analyzed using betulin isolated from *B. platyphylla* bark as an HPLC-DAD analysis standard. The purity of the isolated betulin was about 94% and was detected in 26.4 min at a wavelength of 210 nm in an HPLC-DAD analysis system. Betulin was identified as a major component of *B. platyphylla* bark. (Figure 1A). In addition, for quantitative analysis, the standard product of betulin was serially diluted to 100–2000 μg/mL, and a calibration curve was analyzed. As a result, it showed excellent linearity of 0.99 or higher (Figure 1B). Within the concentration range in which the linearity was confirmed, the analysis was performed with 10 mg/mL of *B. platyphylla* bark extract, and the content of betulin was found to be about 11.8% (Figure 1B).

### 3.2. Effect of Betulin on Human Periodontal Ligament (HPDL) Cell Viability and Confluence

**Figure 2:**
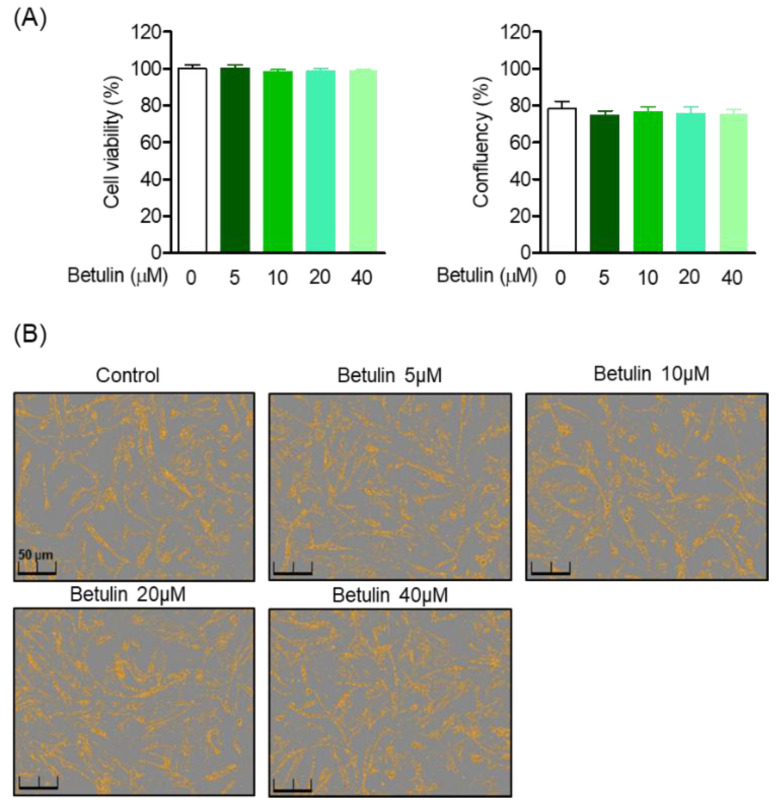
Effects of betulin on HPDL cells cytotoxicity and confluency. HPDL cells were seeded 1 × 10^4^ cell/mL for 24 h, then after treatment with each indicated concentration (5–40 μM) of betulin for 24 h, cell viability was analyzed through MTT assay (**A**). The cell confluents were analyzed using the Incucyte^®^ Live-Cell assay system to marked the cell confluency of normal cells (**B**).

MTT analysis was performed to evaluate the toxicity of betulin isolated from *B. platyphylla* bark on HPDL cells, and the morphology of HPDL cells to betulin was observed. Cell viability was calculated by comparing the ratio of the control cell group to the treated cell group, and the viability of the control group was normalized to 100% and compared. Betulin was treated with HPDL cells at concentrations of 0, 5, 10, 20, and 40 μM for 48 h, and did not show toxicity in the treatment concentration range within 48 h (Figure 2A). In addition, there was no effect on the cell confluence and morphology within the betulin treatment concentration range (Figure 2B).

### 3.3. Effect of Betulin on C/EBP and 11β-HSD1 Activity Regulation in PG-LPS Stimulated HPDL Cells

**Figure 3:**
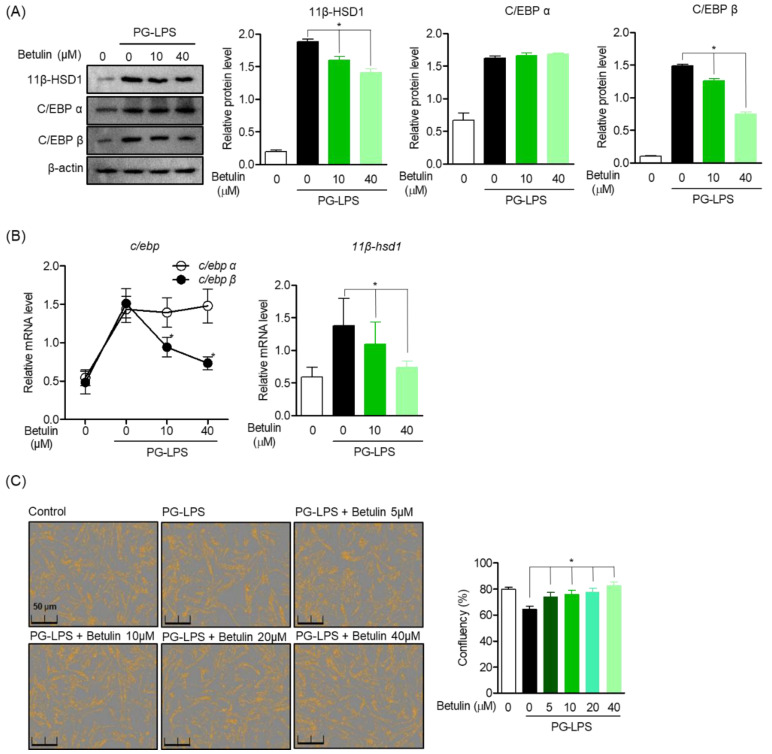
Effect of Betulin on C/EBP and 11β-HSD1 Activity Regulation in PG-LPS Stimulated HPDL Cells. The cells (1 × 10^6^ cells/mL) were pre-treated with 10 and 40 μM for the 6 h then after, PG-LPS (1 μg/mL) was treated for the 24 h. The expression of each proteins (**A**) and gene level (**B**) were analyzed by Western blot and real time pcr. The cell confluents were analyzed using the Incucyte^®^ Live-Cell assay system to marked the cell confluency of normal cells (**C**). * *p* < 0.05 was considered significant differences between each treated groups.

11β-HSD1 regenerates glucocorticoids, amplifies their action, and contributes to the induction of glucocorticoid responsive genes, thereby promoting inflammation. Moreover, it is attributed to the activity of the transcription factor C/EBP. Therefore, Western blot analysis was performed to evaluate the effect of betulin on the regulation of 11β-HSD1 expression through C/EBP activity in HPDL cells stimulated with PG-LPS. As a result, betulin down-regulated the protein expression of C/EBP β increased by PG-LPS in a concentration-dependent manner but did not show an effect on the C/EBP α protein, and it was confirmed that the expression of 11β-HSD1 was also downregulated. (Figure 3A). Moreover, betulin inhibited the gene levels of C/EBP β and 11β-HSD1 increased by PG-LPS, these results suggest that downregulation of C/EBP β by betulin also affects the expression of 11β-HSD1 (Figure 3B). In addition, betulin is concentration-dependently protected HPDL cells lost by PG-LPS. Therefore, these results suggest the possibility of a cytoprotective effect through downregulation of C/EBP β and 11β-HSD1 of betulin (Figure 3C).

### 3.4. Inhibitory Effect of Betulin on Inflammatory Mediators by Regulating 11β-HSD1 and C/EBP Activities

**Figure 4:**
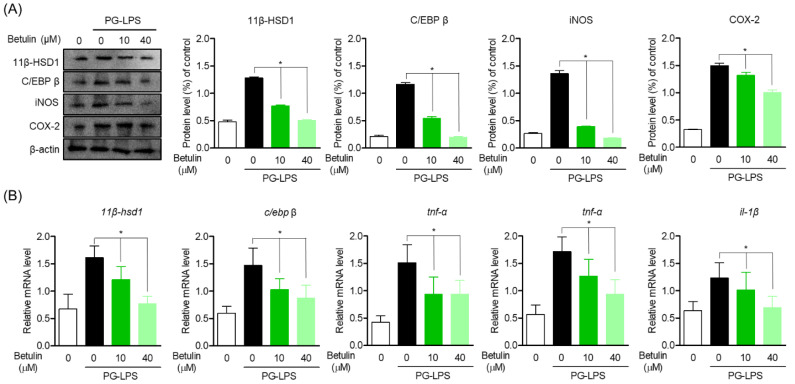
Inhibitory Effect of Betulin on Inflammatory Mediators by Regulating 11β-HSD1 and C/EBP Activities. The cells (1 × 10^6^ cells/mL) were pre-treated with 10 and 40 μM for the 6 h then after, PG-LPS (1 μg/mL) was treated for the 24 h. The expression of each proteins (**A**) and pro-inflamatory cytokine gene level (**B**) were analyzed by Western blot and real time pcr. * *p* < 0.05 was considered significant differences between each treated groups.

In the previous results, betulin effectively suppressed the protein expression and gene levels of C/EBP β and 11β-HSD1 up-expressed by PG-LPS. Therefore, the effect of betulin on the inflammatory mediator and cytokine produced by PG-LPS and the expression of C/EBP β and 11β-HSD1 were also confirmed. In the results of the study, the expression of pro-inflammatory mediator iNOS and COX-2 was increased by PG-LPS stimulation, and accordingly, the expression of C/EBP β and 11β-HSD1 increased together as in the previous results, betulin downregulated C/EBP β and 11β-HSD1 with inhibition of the expression of pro-inflammatory mediators (Figure 4A). In addition, betulin concentration-dependently inhibited the gene expression levels of pro-inflammatory cytokines *tnf-α*, *il-6* and *il-1β* increased by PG-LPS, and at the same time downregulated *11β-hsd1* (Figure 4B). These results suggest that the inhibition of the inflammatory response through 11β-HSD1 inhibition is an important therapeutic strategy in periodontitis as well as in several previously reported metabolic diseases. In addition, these results proved the anti-inflammatory effect of betulin, in vitro, and suggest the possibility that C/EBP β and 11β-HSD1 may be involved in the downregulation of pro-inflammatory mediators and cytokines.

### 3.5. Induction Effect of Betulin on Osteoblast Differentiation through HSD Activity Regulation

**Figure 5:**
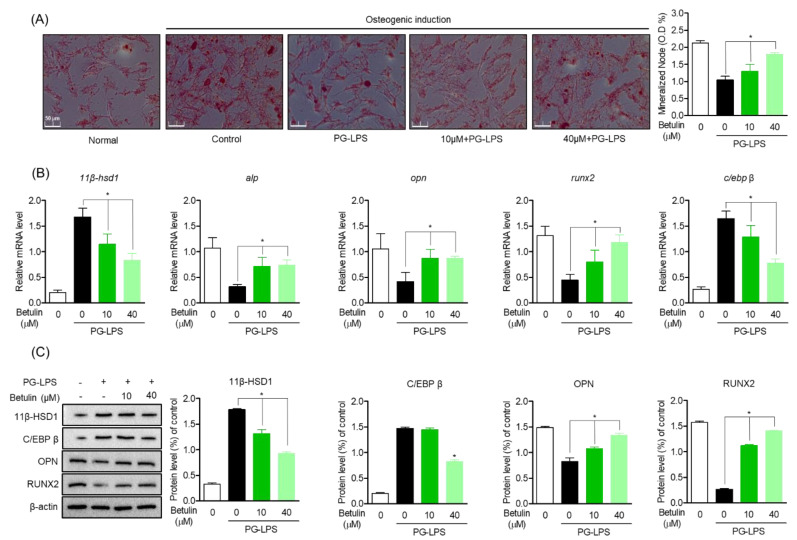
Inhibitory Effect of Betulin on Inflammatory Mediators by Regulating 11β-HSD1 and C/EBP Activities. The HPDL cells (5 × 10^3^ cells/mL) were pretreated with or without the indicated concentration of betulin, after treatment with PG-LPS, it was cultured for 14 days. The result of mineralization was measured by alizarin red s (ARS) staining (**A**). The level of osteogenic induction specific genes (*alp, opn, runx2*) and proteins were analyzed by real-time PCR (**B**) and Western blot (**C**). The results were normalized to *gapdh* and β-actin expression. * *p* < 0.05 was considered significant differences between only PG-LPS treated groups.

To evaluate the effect of betulin on the recovery of lost alveolar bone, one of the important treatment strategies for periodontitis, the effect of betulin on the recovery of osteoblast differentiation capacity of HPDL cells lost by PG-LPS was evaluated. In this study, HPDL cells were induced to differentiate into osteoblasts for 14 days, and the levels of betulin against PG-LPS were evaluated for the mineralization capacity of HPDL cells and the levels of osteoblast differentiation inducing proteins and genes. As a result, it was confirmed that the calcification ability of HPDL cells was increased according to the induction of osteoblast differentiation, and the mineralization ability of HPDL cells lost by PG-LPS treatment was restored again (Figure 5A). Therefore, the levels of the important osteoblast-specific genes *alp*, *opn*, and *runx2* in the process of inducing osteoblast differentiation were also evaluated. The osteoblast-specific gene expression lost by PG-LPS treatment was recovered again by betulin treatment, and the levels of 11β-HSD1 and C/EBPβ were down-regulated (Figure 5B). In addition, these results were similarly confirmed in the protein expression level of the osteoblast-specific gene, betulin down-regulated the proteins of 11β-HSD1 and C/EBP β expressed by PG-LPS, and at the same time restored the expression of osteoblast-specific maker proteins (Figure 5C). These results suggest that betulin is effective in restoring lost alveolar bone as well as inhibiting inflammation, an important treatment strategy for periodontitis. We propose the possibility that botulin is 11β-HSD1 and C/EBP β regulation may be involved in the calcification of HPDL cells lost by PG-LPS.

### 3.6. Protective Effect of Betulin on Alveolar Bone and Fibrous Tissue Lost with PG-LPS in a Periodontitis-Induced In Vivo Model

**Figure 6:**
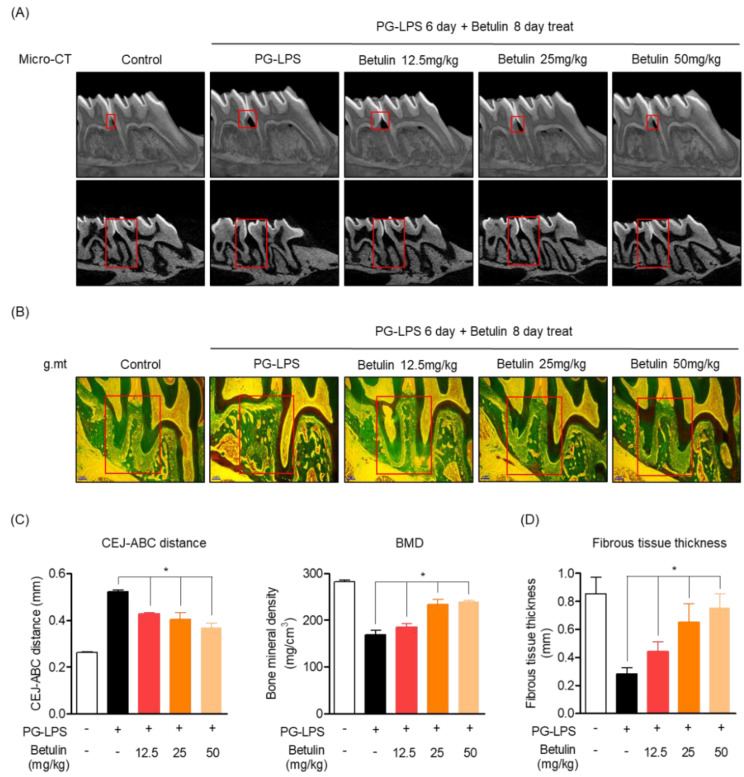
Protective effect of betulin on alveolar bone and fibrous tissue lost with PG-LPS in a periodontitis-induced in vivo in vivo model. Methods of inducing periodontitis in vivo in vivo models are described in materials and methods Section 2.9. The images of the alveolar bone newly formed by betulin were taken through micro-CT (3 mm) (**A**). Confirmation of periodontal fibrous tissue protective effect through Goldner’s Masson Trichrome staining (**B**). The CEJ-ABC distance and BMD were quantified through VGStudio MAX 1.2.1 software by setting the red part as the quantitative area (**C**). Measurement of the thickness of periodontal fibrous tissue by setting the red part as the quantitative area (**D**). * *p* < 0.05 was considered significant differences between only PG-LPS treated groups. Each group (*n* = 3).

In order to support the claim of botulin is alveolar bone protective effect in the PG-LPS-induced in vitro periodontitis induction model, the efficacy was demonstrated in a rat model in which periodontitis was induced by PG-LPS injection. The eight-week-old Sprague-Dawley rat was injected with PG-LPS for six days between the first and second molars of the right maxilla, followed by oral injection. The degree of alveolar bone loss was confirmed using micro-CT by oral administration of betulin at the indicated concentration for 8 days and injection of PG-LPS once every two days. As a result, in the group treated with PG-LPS alone, the periodontal crest was loosened by periodontitis, and the recovery of the periodontal crest loosened in a concentration-dependent manner by administration of betulin could be confirmed through 3D-Micro-CT. In addition, it was proven through 2D-Micro-CT analysis that betulin is effective in the recovery and protection of the alveolar bone lost by PG-LPS. In addition, the length of the periodontal crest loosened by PG-LPS was quantified. In the group treated with PG-LPS alone, the length of the periodontal crest was 0.53 mm, but it was confirmed that the length of the periodontal crest was reduced to 0.32, 0.31, and 0.25 mm by the concentration-dependent administration of betulin. Additionally, betulin showed the effect of restoring the lost bone mineral density (Figure 6A,C). Likewise, betulin showed a protective effect by restoring the periodontal fibrous tissue damaged by PG-LPS in a concentration-dependent manner, and also restored the thickness of the defective periodontal fibrous tissue between the missing maxilla 1st and 2nd molars in a concentration-dependent manner (Figure 6B,D).

These results suggest that betulin has the effect of recovering alveolar bone and fibrous tissue lost by PG-LPS not only in the in vitro periodontitis-inducing model shown above, but also in the in vivo periodontitis inducing model.

### 3.7. Inhibitory Effect of Betulin on Periodontal Tissue Infiltration in PG-LPS-Induced Periodontitis In Vivo Model

**Figure 7:**
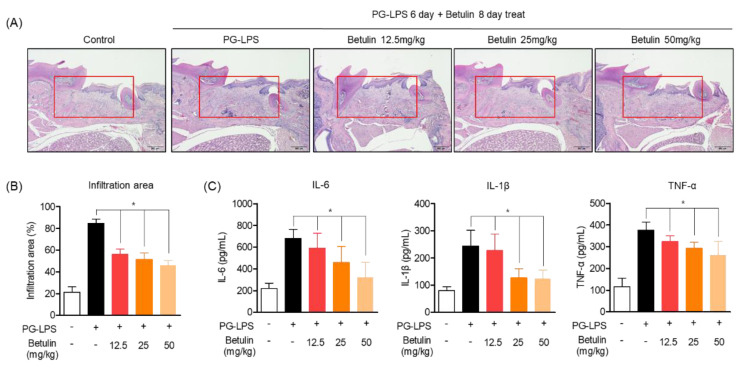
Inhibitory effect of betulin on periodontal tissue infiltration in PG-LPS-induced periodontitis in vivo model. Periodontal tissue infiltration due to periodontitis was analyzed by hematoxylin and eosin (H&E) (**A**) staining and is described in materials and methods Section 2.11. The quantification of infiltration was expressed as a percentage (%) of the infiltrating area from the total area of the marking (red area) (**B**). Measurement of pro-inflammatory cytokines using ELISA kit from serum of rats induced with periodontitis by PG-LPS (**C**). * *p* < 0.05 was considered significant differences between only PG-LPS treated groups. Each group (*n* = 3).

In the previous results, the alveolar bone lost by PG-LPS of betulin was recovered in a concentration-dependent manner. Therefore, the effect of betulin on the infiltration of periodontal tissue due to periodontitis induction was confirmed through H&E staining. The in vivo model of periodontitis induced by PG-LPS used the same method as specified in material and methods Section 2.7, and the degree of infiltration of periodontal tissue due to periodontitis was confirmed by photographing the periodontal tissue obtained after the end of the experiment. In the experimental results, it was confirmed that severe invasion of periodontal tissue was induced in the group treated with PG-LPS alone, and the degree of infiltration was recovered in a concentration-dependent manner by treatment with botulin (Figure 7A). In addition, as a result of quantifying the area of the infiltrated tissue, an average of 21.05% of the infiltrating area was confirmed in the normal group, and it was confirmed that the invasion area was increased to 84.67% by PG-LPS treatment. Moreover, the tissue infiltration area was reduced to 56.10, 51.46 and 45.72% by concentration-dependent treatment of betulin, respectively (Figure 7B). Therefore, in order to confirm the evidence for the inhibition of inflammatory infiltration, the production of the representative proinflammatory cytokines IL-6, IL-1, and TNF of periodontitis in serum isolated from the blood of periodontitis-induced rats was evaluated. As a result, oral administration of betulin showed the effect of down-regulating the production level of inflammatory cytokines increased by PG-LPS (Figure 7C). These results suggest that betulin not only protects the alveolar bone in the in vivo model of periodontitis induction, but also show anti-inflammatory effects on tissue infiltration due to inflammation. This is the result of demonstrating the claim of anti-inflammatory, alveolar bone induction and the protective effect in the in vitro periodontitis induction model.

## 4. Discussion

11β-HSD1 is an isoenzyme that catalyzes the interconversion of hormone inactive and active glucocorticoids, a close relationship between increased glucocorticoids and chronic periodontitis has been reported, these reports indicate that periodontitis has a close relationship with the increased expression of 11β-HSD1 [26,27]. In addition, according to several previously reported studies, 11β-HSD1 is a major regulator of GC activity in bone tissue, and excessive 11β-HSD1 expression is known to cause severe damage to joint tissue along with tissue inflammation and bone loss at the same time [28]. In several previously reported studies, betulin inhibited the decomposition of articular cartilage through anti-inflammatory effects by stimulation of LPS or IL-1 and down-regulation of MMP-3 and MMP-13 expression and activity, its efficacy has been proven [29,30]. However, the relationship between betulin and periodontitis and 11β-HSD1 has not been identified. Therefore, in this study, we evaluated the effect of betulin on CEBP, an important regulator of 11β-HSD1 expression in a periodontitis-inducing in vitro model. In addition, the in vivo periodontitis induction model also demonstrated the efficacy of betulin in inhibiting periodontitis, which is an important treatment strategy for periodontitis, and recovering loss alveolar bone. In the results of this study, first, the content of betulin standardized from bark of *B. platyphylla* extract was identified for use in the study, and it was confirmed that the content was about 27.1%. Therefore, as a result of comparing the content of betulin in the birch extract in the previously reported literature, it was found that the level of the content was similar [31].

Betulin isolated from *B. platyphylla* did not show toxicity in human periodontal ligament (HPDL) cells and inhibited the expression of transcription factor C/EBP protein, in a concentration-dependent manner, PG-LPS-stimulated HPDL cells inhibited the expression of transcription factor C/EBP protein with 11β-HSD1 and protected HPDL cells lost with PG-LPS. These results suggest that betulin regulates the transcription factor C/EBP β related to the expression of 11β-HSD1 by PG-LPS, thus suggesting the potential for previously reported anti-inflammatory effects as well as the potential for anti-inflammatory effects in periodontitis [32]. In periodontitis, inflammation-inducing factors, such as TNF-α, IL-1β, and IL-6, are secreted from PDL cells due to bacterial endotoxin release, causing periodontal inflammation and infection [33]. Therefore, as a result of evaluating the effect of betulin on the production and expression of TNF-α, IL-6, and IL-1β, which are known as pro-inflammatory mediators and representative inflammatory cytokines of periodontitis in PG-LPS-stimulated HPDL cells, betulin downregulates the protein production of 11β-HSD1 and transcription factor C/EBP β together with inhibition of iNOS and COX-2 proteins produced by PG-LPS, and 11β-HSD1 with increased pro-inflammatory cytokines. Moreover, the gene level of C/EBP β was down-regulated in a concentration-dependent manner. These results show a close relationship of betulin to the previously reported effect of inhibiting periodontitis by the regulation of 11β-HSD1 in chronic periodontitis [34]. In addition to its anti-periodontitis effect, betulin has been suggested for the recovery of the alveolar bone lost due to inflammation, which is another important treatment strategy for periodontitis [35]. Therefore, in this study, as a result of evaluating the effect of betulin on osteoblast differentiation ability of HPDL cells inhibited by PG-LPS, it was confirmed that betulin promotes osteoblast differentiation of lost HPDL cells. In addition, to demonstrate the anti-inflammatory effect of betulin stimulated with PG-LPS and the recovery effect of lost osteoblast differentiation, the efficacy of betulin in the in vivo periodontitis-induced rat model was evaluated. Betulin recovered the lost alveolar bone mass along with the length of the crest of the alveolar bone loosened by the induction of periodontitis, in addition to the recovery effect of the periodontal tissue infiltrated by PG-LPS. In addition, the production amount of important inflammatory cytokines in periodontitis, such as the aforementioned TNF-α, IL-6, and IL-1β, was also effectively down-regulated.

## 5. Conclusions

In this study, it was confirmed that betulin was involved in periodontitis inhibition and the restoration of lost alveolar bone in both in vivo and in vitro models of periodontitis induction. In particular, betulin has demonstrated the effect of effectively suppressing the expression of major inflammatory cytokines in periodontitis and restoring the lost alveolar bone. The pharmacological validity of betulin was investigated for the suppression of periodontal inflammation and the protective effect of alveolar bone, two important treatment strategies for periodontitis. Therefore, a new pharmacological activity for betulin has been newly confirmed, and the potential as a therapeutic agent for periodontitis derived from natural products is suggested.

## Figures and Tables

**Table 1:** Primer sequences.

Target Gene	Sequence (5′→3′)	
* 11β-hsd1 *	Forward	GGGGTACCTTTTTCCCCGCTCTACTGATAACT
	Reverse	TTCTCGAGCCGACAGGGAGCTGGCCTGAAGACT
* c/ebp β *	Forward	AGAAGACCGTGGACAAGCACAG
	Reverse	CTCCAGGACCTTGTGCTGCGT
* c/ebpα *	Forward	CGGACTTGGTGCGTCTAAGATG
	Reverse	GCATTGGAGCGGTGAGTTTG
* il-6 *	Forward	AGTGAGGAACAAGCCAGAGC
	Reverse	GTCAGGGGTGGTTATTGCAT
* il-1β *	Forward	AACCTCTTCGAGGCACAAGG
	Reverse	GTCCTGGAAGGAGCACTTCAT
* tnf-α *	Forward	GCCTCTTCTCCTTCCTGATCGT
	Reverse	TGAGGGTTTGCTACAACATGGG
* alp *	Forward	TGCAGTACGAGCTGAACAGG
	Reverse	GTCAATTCTGCCTCCTTCCA
* opn *	Forward	TCAGCTGGATGACCAGAGTG
	Reverse	TTGGGGTCTACAACCAGCAT
* runx2 *	Forward	TCTTAGAACAAATTCTGCCCTTT
	Reverse	TGCTTTGGTCTTGAAATCACA
* gapdh *	Forward	TGTTCGTCATGGGTGTGAAC
	Reverse	GTCTTCTGGGTGGCAGTGAT

## Data Availability

Data sharing is not applicable to this article.
